# Evaluation the quality of bag-mask ventilation by E/C, T/E and hook technique (a new proposed technique)

**DOI:** 10.1186/s12871-023-02349-w

**Published:** 2023-11-23

**Authors:** Moloud Balafar, Mahboub Pouraghaei, Seyed Pouya Paknezhad, Saba Nemati Ahmad Abad, Hassan Soleimanpour

**Affiliations:** 1 Emergency and trauma care research center, Tabriz University of Medical Sciences, Tabriz, Iran; 2 Student Research Committee, Tabriz University of Medical Sciences, Tabriz, Iran; 3 Road Traffic Injury Research Center, Tabriz University of Medical Sciences, Tabriz, Iran

**Keywords:** Bag -mask ventilation, Airway management, E/C method, Thenar eminence technique, Hook method

## Abstract

**Background:**

Bag-Mask Ventilation (BMV) is a crucial skill in managing emergency airway situations and induction of general anesthesia. Ensuring proficient BMV execution is imperative for healthcare providers. Various techniques exist for performing BMV. This study aims to compare the quality of ventilation achieved using the E/C technique, Thenar Eminence (T/E) technique and a novel approach referred to as the hook technique. The goal is to identify the most effective single-person BMV method.

**Method:**

We conduct a pilot study on manikins involving 63 medical staff members who used the hook technique for ventilation. Subsequently, we obtained ethical approval and patient guardian consent to perform the study on 492 emergency department (ED) patients. These patients were randomly divided into three groups, with each group subjected to one three ventilation techniques. The study focused on patients requiring reliable airway management for rapid sequence intubation (RSI). Ventilation was administrated using bag-mask device connected to the capnograph. End-tidal CO2 (ETCO2) levels were recorded. Demographic data were collected and analyzed by SPSS software version 22. Success rates were reported as frequency (percentage) as well as mean ± standard deviation.

**Result:**

Comparing partial pressure of CO2 (PCO2) results obtained via capnography between T/E, E/C and hook techniques, we found that the successful ventilation rate was 87.2% for T/E, 89.6% for E/C, and 93.3% for the hook methods. The hook method demonstrated significantly higher success rate compared to the other two techniques (P-value = 0.038). Furthermore, we observed statistically significant trends in PCO2 changes between measurements both within and between groups (P-value < 0/001).

**Conclusion:**

Our study indicates that the hook method achieved notably higher success rate in ventilation compared to the T/E and E/C methods. This suggests that the hook method, which involves a chin lift maneuver while securely fitting the mask, could serve as a novel BMV technique, particularly for resuscitation with small hands for a prolonged use without fatigue and finger discomfort. Our finding contributes to the development of a new BMV method referred to as the hook technique.

**Trial registration:**

IRCT registration number: IRCT20121010011067N5. URL of trial registry record: https://www.irct.ir/trial/57420.

## Introduction

**Fig. 1:**
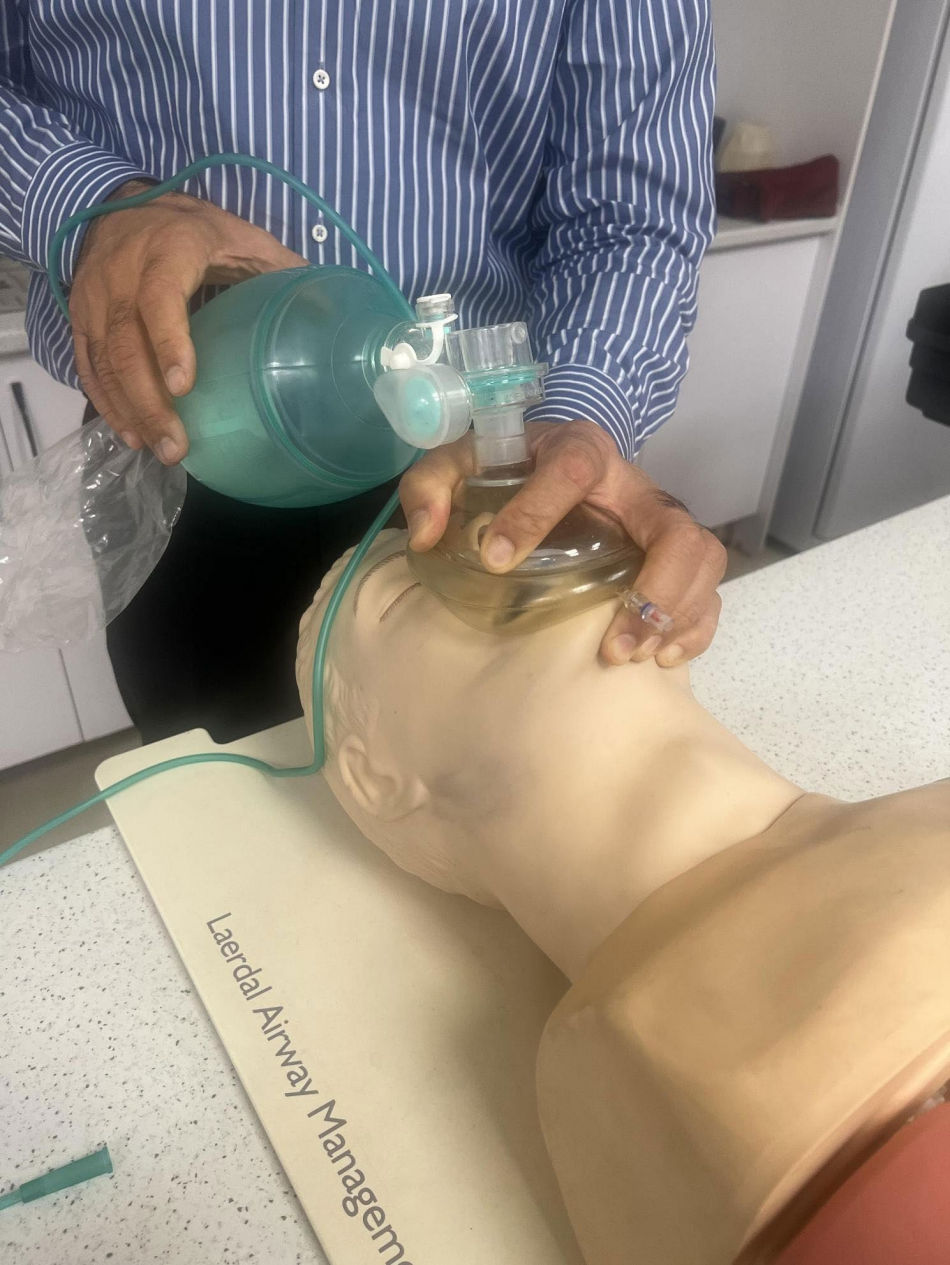
E/C technique application in a manikin

**Fig. 2:**
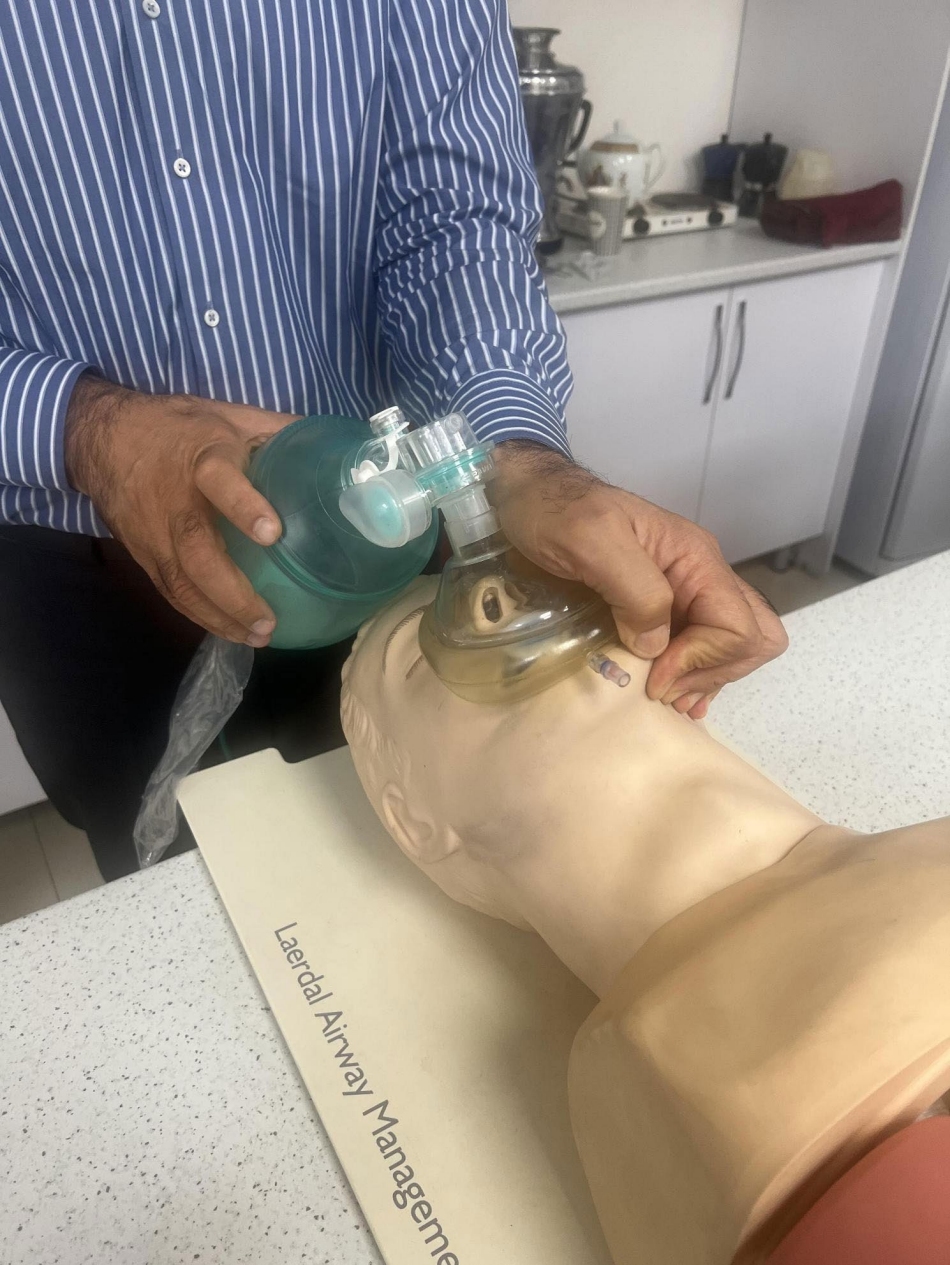
Thenar Eminence (T/E) technique application in a manikin

**Fig. 3:**
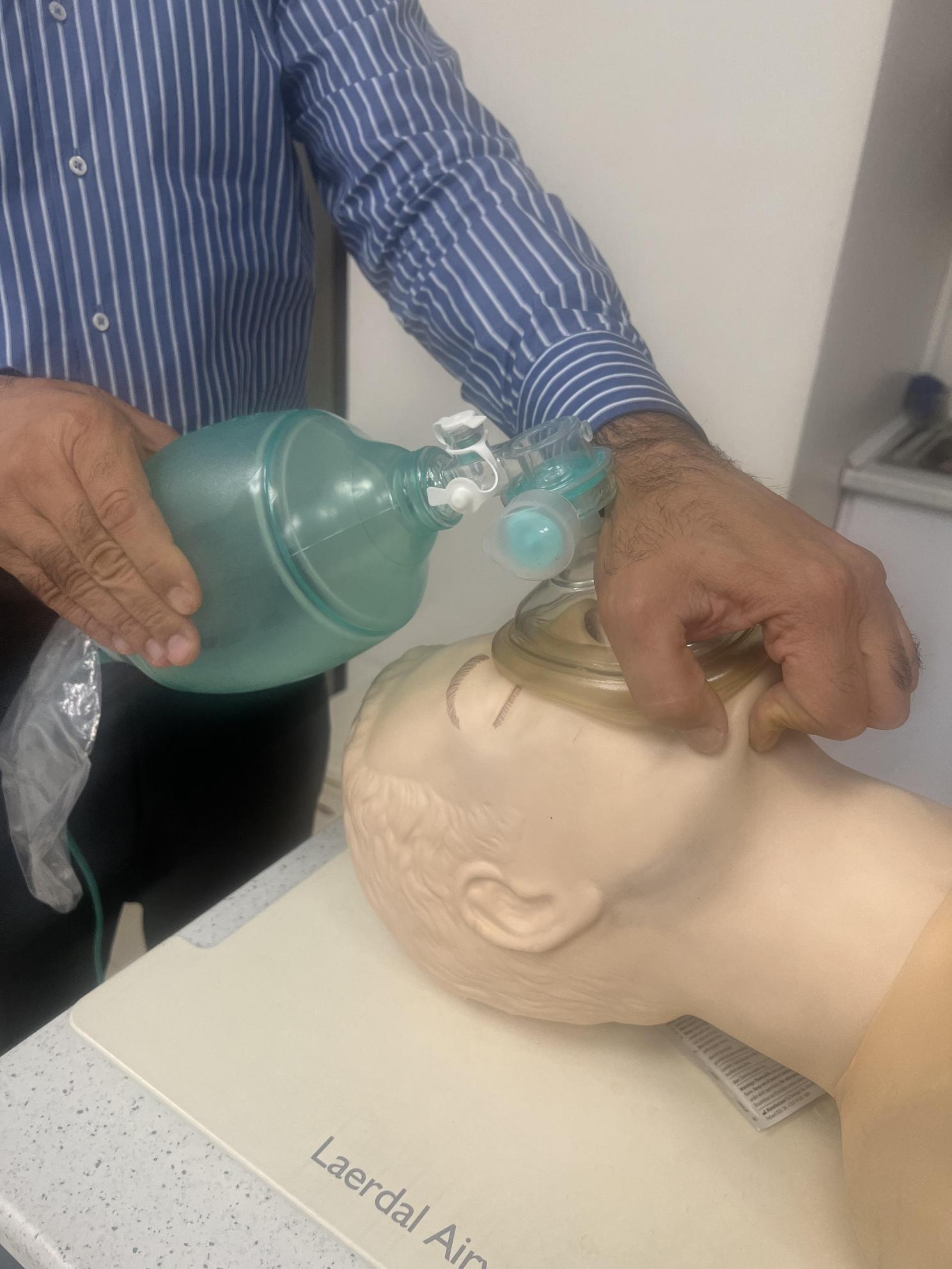
Hook technique application in a manikin

Bag-Mask Ventilation (BMV) plays a pivotal role in managing emergency airway situations and facilitating general anesthesia [1]. Despite its apparent simplicity, BMV demands precision and expertise. It requires skilled operator who can securely position the mask and administer the requisite positive pressure [2]. Inexperienced use of BMV is considered a relative contraindication [3]. According to the American Society of Anesthesiologists (ASA), Difficult Bag-mask Ventilation (DMV) occurs when an anesthesiologist struggle to establish effective BMV due to various issues, including incomplete mask sealing, gas leakage around the mask, high resistance to gas inlet and outlet, and signs of poor ventilation leading to a drop in Oxygen saturation below 90%, even with 100% oxygen [3]. It is commonly recommended that BMV during cardio-pulmonary resuscitation (CPR) be performed by two experienced operators, given the challenges faced during single-person ventilation (4). However, scenarios may arise where only one healthcare provider is available to administer BMV effectively. Hence, it is essential to identify a technique that can be performed solo. Multiple BMV techniques exist, one of which is the E/C technique, involving placing the mask pad between the thumb and index finger while using the other fingers to perform a chin lift maneuver [5]. Another technique known as thenar eminence (T/E) technique is widely recognized [6]. In this study, we introduced a novel BMV technique referred to as the hook method, which has not been previously described. Given the critical importance of BMV and necessity of learning and refining these techniques, our research aims to compare the quality of BMV using the E/C, T/E and hook techniques to determine the most effective single-person BMV method. Figures 1, 2 and 3 show these mentioned BMV techniques.

## Materials and methods

To design this study, we initially conducted a pilot study on manikins with participation of 63 emergency medicine residents who were eligible for airway management.

This pilot study aimed to evaluate the effectiveness of the hook method, a technique involving the resuscitator’s palm on the mask pads front and fingers guiding the patients chin upwards. This approach was subsequently compared to the E/C and T/E techniques. A divider was used to ensure that the recorder assistant remined blinded to the BMV technique employed. Chest rises quality was recorded for all three techniques. Upon successful outcomes from the manikin pilot study, and after obtaining ethical approval and consent from patient’s guardians, we proceeded to enroll 492 patients admitted to ED. Sample size was calculated based on pilot study considering a power of 80% and a type one error of less than 0.05. These patients were randomly divided into three groups using http://www.randomization.com site, each subjected to one of the three ventilation techniques. The study focused on patients requiring reliable airway management with an indication for rapid sequence intubation (RSI). Patients who required intubation using other methods, obese patients, those with facial anomalies, a history of malignant hyperthermia, a nasogastric tube, significant burns, peritonitis lasting more than three days, and patients with extensive beards and mustaches were excluded from the study.

**Fig. 4:**
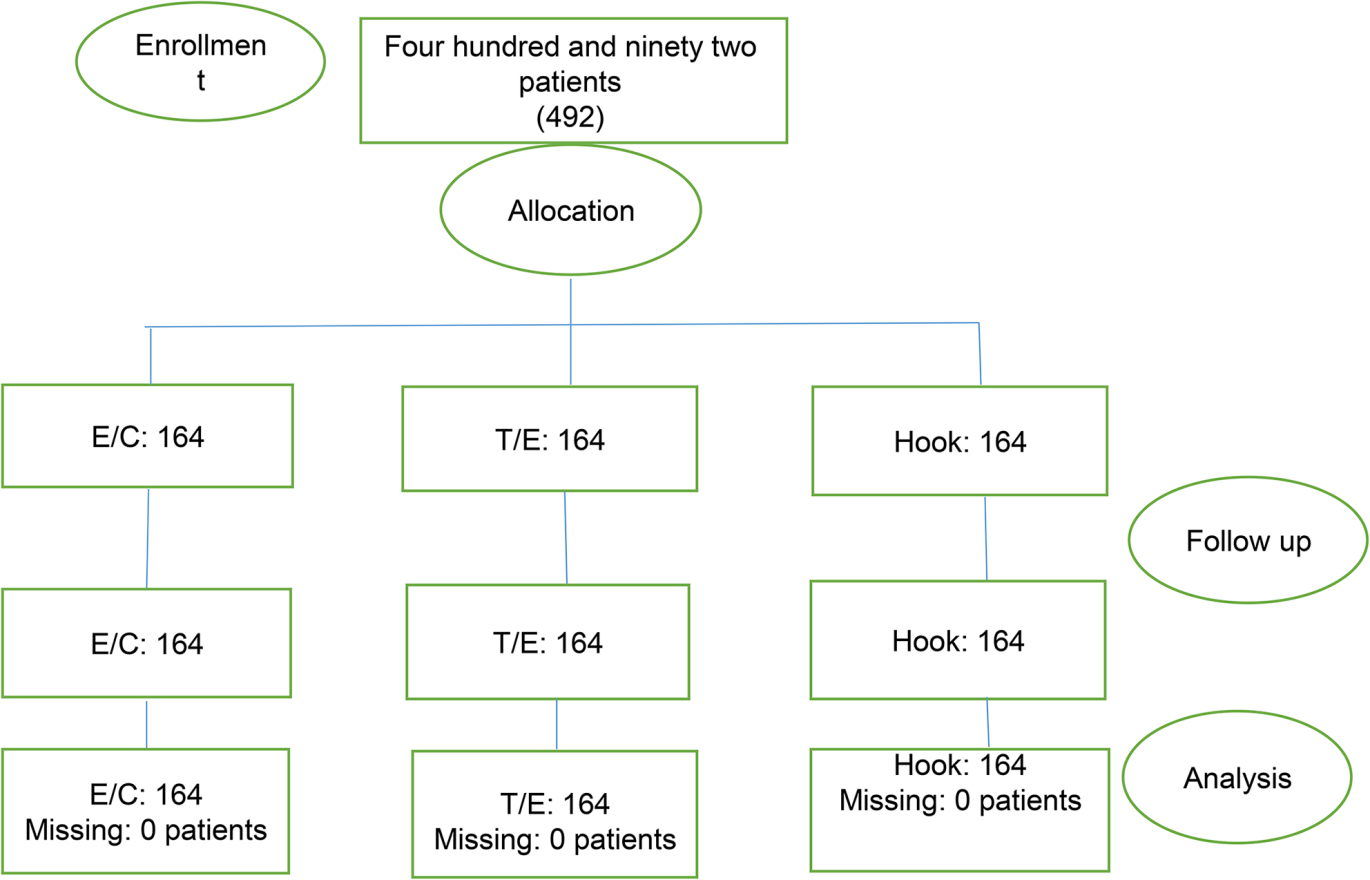
Flow chart of the study

After the screening, pre-oxygenation, premedication, and induction of anesthesia, patients were ventilated by a bag-mask attached to a capnograph by an emergency medicine specialist, senior resident, or emergency attending. Twelve breaths/min with 100% FIO2 were given. We used the RESPIRONICS brand capnograph device, the product of Respironics, Inc. with specifications of Cosmas Court 2271, Carlsbad, CA USA 92,009 was used. Capnography device is a standard calibrated device and its gold standard for improving ventilation with Bag - Mask. The ETCO2 sensor placed between mask’s inlet and bag’s connector part (outlet). Depending on whether patients were in group I, II or III, before induction of anesthesia, patients were ventilated 8 times by bag-mask and at the end of each ventilation, ETCO2 was recorded. BMV success assessment is defined as follows: Increase ET CO2 to more than 20 mmHg and return to baseline [6]. Comparison between Bag-Mask ventilation using E / C, T/E and Hook methods in the three study groups was performed. Flowchart of the study is available in the Fig. 4. We used the opaque drape over patient to separate the mouth and nose from the chest. It was impossible for two assessors (professors of emergency medicine) to see which BMV techniques were used by an emergency medicine specialist, senior resident, or emergency attending. On the other side of the curtain, two evaluators (two emergency medicine specialists) evaluated the BMV techniques based on the chest expansion score from 1 to 4. To evaluate Chest Rising in the studied patients, by visual method, patients were categorised into four groups: the amount of chest rising in group one was between 0 and 25% (score 1), 25–50% in group two (score 2), 50–75% in group three (score 3) and 75–100% in group four (score 4). Patients in group one and two considered to have unsuccessful ventilation due to lack in chest rise, and in groups three and four, the ventilation was considered successful.

### Data analysis

Data analysis was conducted using SPSS software version 22. We collected and analyzed demographic data, reporting success rates as frequencies and mean ± standard deviation.

Success and failure in the Pilot study were expressed as a percentage and the results were reported as frequency (percentage) as well as.

We utilized statistical tests such as Kolmogorov-Smirnov test to assess data normality, the Kruskal-Wallis test for non-normally distributed data, and repeated measure tests. Additionally, we calculated Diagnostic test parameters including positive and negative predictive values, sensitivity, specificity, and Youden´s Index. A P-value of ≤ 0.05 was considered statistically significant.

## Results

Results of pilot study showed 93.7% success rate in ventilating manikins for hook method, compared to a 90% and 87.3% success rate for E/C and T/E methods respectively. No significant difference has been found between three methods.

In patients ventilated by the hook method, 47.6% were women and 52.4% were men. The mean age of patients was 46 ± 17.4 with a minimum age of 18 years and maximum age of 99 years. In patients ventilated by the E/C technique, 48.2% of women and 51.8% of men. The mean age of patients in this group was 50 ± 18.8 with a minimum age of 18 and a maximum age of 99 years. In T/E method group 48.2% of patients were female and 51.8% were male. The mean age of patients in this group was 47 ± 17.6 with a minimum age of 18 and a maximum age of 89 years.

There was no significant difference in terms of sex distribution between three groups (PV: 0.998). In comparison with PCO2 results obtained from capnography between E/C, T/E and Hook techniques, the ventilation rate was 87.2% and 89.6% in the T/E and E/C methods respectively and 93.3% in the Hook method. The success rate of ventilation with the Hook method was considerably higher than the E / C and T/E methods. There was a significant difference in ventilation success between the three methods (PV = 0.038).

PCO2 measurement times from the 2nd to the 10th second were recorded in three techniques and a statistically significant difference was observed in fifth to eighth seconds (PV = 0.008), (PV < 0/001), (PV < 0/001) respectively.

**Fig. 5:**
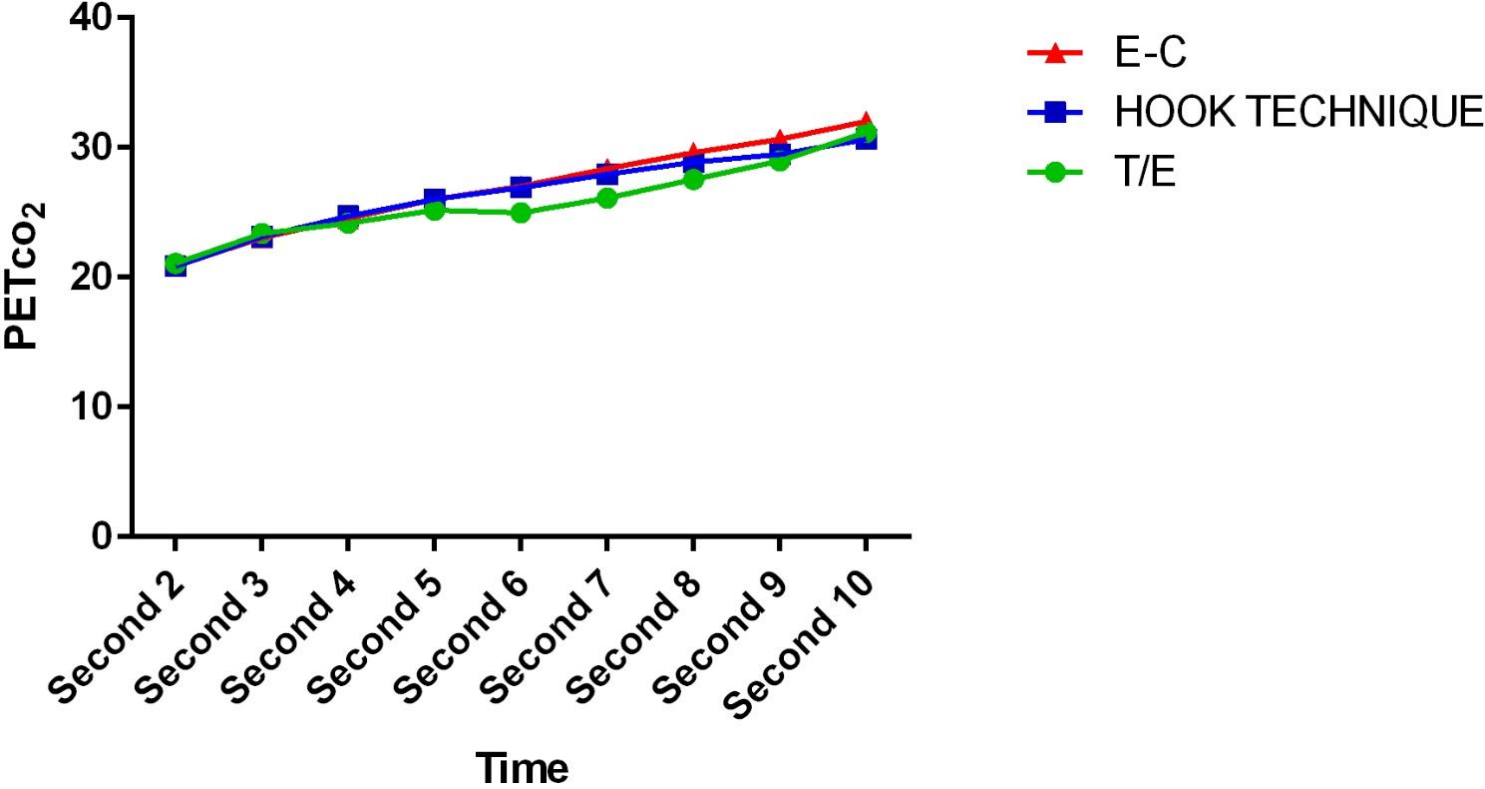
Trend of Pco2 changes at measurement times by E / C, T/E and hook methods

More information is shown in Table 1. In the comparison between groups, the trend of PCO2 changes in different measurements was statistically significant (pv = 0.006). Also, the within-group comparison of PCO2 changes in different measurements was statistically significant (P < 0/001). Figure 5 shows the trend of PCO2 changes at measurement times by E / C, T/E and hook methods. The sensitivity of chest rising in all three techniques was 100%. Specificity in E/C technique, T/E technique and hook method was 100%, 28.6% and 50% respectively. Positive predictive value was 100% in E/C technique, 90.5% in T/E technique and 96.5% in hook method. Negative predictive value was 100% in all three groups.

**Table 1:** Comparison of capnograph-derived PCO2s in different measurements between ventilated patients with hook method, T/E method and E/C technique

Measurement time	Technique used for ventilation			p-value	Group effect p-value	Time effect p-value
	T/E method Median (IQR*)	E/C method Median (IQR)	Hook method Median (IQR)			
The 2nd second	20 (20–23)	21(22 − 20)	21 (20–22)	0.199	0.006**	< 0.001**
The third second	24 (22–25)	23(24 − 21)	23 (23–24)	0.114		
The fourth second	24 (22–26)	25(26 − 23)	25 (24–26)	0.071		
**The fifth second**	**26(24–27)**	**26(28 − 24)**	**26 (25–27)**	**0.008**		
**The sixth second**	**25 (23–27)**	**28(29 − 25)**	**27 (26–28)**	**< 0.001**		
**The seventh second**	**26(24–29)**	**29(30 − 27)**	**28 (27–29)**	**< 0.001**		
**The eight second**	**28(26–30)**	**30(31 − 29)**	**29 (28–30)**	**< 0.001**		
The ninth second	29 (25.27-31)	31(33 − 29)	30 (28–31)	0.134		
The tenth second	31(29–34)	32(34 − 30)	31 (29–32)	0.377		

*IQR = InterQuartileRange

** significant

## Discussion

Bag-Mask ventilation is one of the essential skills that every physician should know how to do it properly. Since there are several different ways to BMV, it is important to know the best way to do it that is both convenient and effective by single person. We introduced a method called hook method in which the resuscitator’s palm will be used on the front of the mask pad and his or her fingers will guide the patient’s chin upwards, And we compared this method with the E/C and T/E techniques which are common and valid. If the hook-method is successful, it can be a good alternative to do BMV.

In a study done by Umesh et al. about comparing the E/O technique to the E/C technique, they concluded that the E-O technique is superior to the E-C technique in manikins during single person bag-mask ventilation performed by novices. experienced resuscitators, E/C provided a proper chest expansion. If one technique fails, another can be used instead [5].

Soleimanpour M et al., evaluated the quality of four different BVM ventilation techniques – E-C, Thenar Eminence, Thenar Eminence (Dominant hand)-E-C (Nondominant hand), and Thenar Eminence (Nondominant hand)-E-C (Dominant hand) – among two novice and experienced groups. They concluded that Novices did Thenar Eminence (non-dominant hand) and E-C (dominant hand) technique better than the other two techniques. Therefore, for new trainees, these two techniques are recommended because of convenience [7].

In another study, Soleimanpour M, et al., compared three techniques on Facility of Bag-Mask Ventilation: Thenar Eminence, E-O, and E-C. They stated that in professional operators there wasn’t any significant difference between these three methods, but in novices, the E/O technique is done better and it’s a good technique to teach to medical students [8].

Osiński et al. showed a better success rate for a behind the head position in bag mask ventilation compared to ventilation from the side [9]. In our study all patients ventilated from behind the head and we did not studied ventilation from the side. Studying the hook method in a side position can help to draw more accurate conclusions about this technique.

In present study, in pilot study on manikin using hook method, the succession rate was 93.7% and 6.3% were unsuccessful. In comparison of PCO2 results obtained from capnography between two E / C, T/E and hook techniques of patients, the success rate of ventilation in patients ventilated with E/C and T/E methods was 87.2% and 89.6% respectively, while in patients ventilated by hook method success rate was 93.9%. In fact, the success rate of ventilation with hook method is somewhat higher than the E / C and T/E methods and in comparing the success rate of ventilation between the three methods, a statistically significant difference was observed (p = 0.038).

### Limitations

This study included 492 patients, which may be considered a relatively small sample size for drawing definitive conclusions. While our findings provide valuable insights, larger and more diverse populations could enhance the generalizability of our results. Our study conducted initial assessments on manikins before progressing to human patients. While manikins offer controlled environments for preliminary data collection, they may not fully replicate the complexities of real-life clinical scenarios, and the applicability of our findings to actual patients should be interpreted with this in mind. Our research was conducted at a single medical center, potentially limiting the diversity of patient demographics and clinical scenarios encountered. Future multi-center studies could provide a broader perspective on the applicability of our proposed bag-mask ventilation technique. Visual observations, such as measuring chest rise, are inherently subjective and susceptible to observer bias. While efforts were made to minimize bias, the possibility of subjective assessments affecting our results cannot be entirely eliminated. This study was conducted in a specific region and among a particular population, which may limit the generalizability of our findings to other ethnic or regional groups with distinct characteristics. We studied only dominant hand in all three techniques. Studying these techniques both with dominant and non-dominant hand can give a better view of their performance in real practice.

## Conclusion

In the present study, the success rate of BMV by hook method in manikins was 93.7%. In the study on patients, the success rate of BMV by hook technique was 93.9 vs. 87.2% compared to the E/C technique. The results expressed that due to the chin lift maneuver while the mask is fixed on the face, for resuscitators with small hands and for long-term use without fatigue and finger pain, this method can be suggested as a new Bag-Mask Ventilation method. This study leads to the development of a new method for bad-mask ventilation called the hook method.

## Data Availability

The datasets generated during and analysed during the current study are not publicly available due to restriction of ethic committee of Tabriz University of Medical Sciences but are available from the corresponding author on reasonable request.

## List of abbreviations

ED: Emergency Department
BMV: Bag-Mask ventilation
DMV: Difficult Bag-mask Ventilation

## References

[1] Soleimanpour H, Sarahrudi K, Hadju S, Golzari SEJ (2012). How to overcome difficult-bag-Mask-Ventilation: recent approaches. Emerg Med.

[2] Weiss AM, Lutes M. Focus on-bag-valve mask ventilation. ACEP News September. 2008 Sep.

[3] El-Orbany M, Woehlck HJ. Difficult Mask Ventilation. Anesthesia & analgesia. 2009;109:1870–1880. 10.1213/ANE.0b013e3181b5881c 19923516

[4] Morrison LJ, Deakin CD, Morley PT, Callaway CW, Kerber RE, Kronick SL, Lavonas EJ, Link MS, Neumar RW, Otto CW, Parr M, Shuster M, Sunde K, Peberdy MA, Tang W, Hoek TL, Bo ¨ttiger BW, Drajer S, Lim SH, Nolan JP, Advanced Life Support Chapter Collaborators. Adult advanced cardiovascular life support: 2010 American Heart Association guidelines for Cardiopulmonary resuscitation and emergency cardiovascular care. Circulation. 2010;122:S729–67. 10.1161/CIRCULATIONAHA.110.970988 20956224

[5] Umesh G, Krishna R, Chaudhuri S (2014). E-O technique is superior to E-C technique in manikins during single person bag mask ventilation performed by novices. J Clin Monit Comput.

[6] Conlon NP, Sullivan RP, Herbison PG, Zacharias M, Buggy DJ (2007). The effect of leaving dentures in place on bag-mask ventilation at induction of general anesthesia. Anesth Analg.

[7] Soleimanpour M, Rahmani F, Ala A, Morteza Bagi HR, Mahmoodpoor A, Golzari SE, Zahmatyar F, Mehdizadeh Esfanjani R, Soleimanpour H (2016). Comparison of four techniques on facility of two-hand bag-valve-mask (BVM) ventilation: E-C, Thenar Eminence, Thenar Eminence (Dominant hand)-E-C (non-dominant hand) and Thenar Eminence (non-dominant hand) - E-C (dominant hand). J Cardiovasc Thorac Res.

[8] Soleimanpour M, Rahmani F, Morteza Bagi HR, Ala A, Mahmoodpoor A, Hassani F, Mahdi Sharifi S, Mehdizadeh Esfanjani R, Soleimanpour H (2018). Comparison of three techniques on facility of bag-mask ventilation: Thenar Eminence. E-O and E-C Anesth Pain Med.

[9] Osiński H, St Cyr A (2019). Comparison of resuscitation techniques using pocket-mask in tactical medicine. Crit Care Innov.

