# Correction: Evaluation the quality of bag-mask ventilation by E/C, T/E and hook technique (a new proposed technique)

**DOI:** 10.1186/s12871-024-02545-2

**Published:** 2024-04-25

**Authors:** Moloud Balafar, Mahboub Pouraghaei, Seyed Pouya Paknezhad, Saba Nemati Ahmad Abad, Hassan Soleimanpour

**Affiliations:** 1 Emergency and trauma care research center, Tabriz University of Medical Sciences, Tabriz, Iran; 2 Student Research Committee, Tabriz University of Medical Sciences, Tabriz, Iran; 3 Road Traffic Injury Research Center, Tabriz University of Medical Sciences, Tabriz, Iran

**Correction:** **BMC Anesthesiol 23, 384 (2023)**

**https://doi.org/10.1186/s12871-023-02349-w**

 Following publication of the original article [1], the authors reported an error found in figure 4 and an error under Materials and methods section.

In figure 4 (Flowchart of the study) the “E/O” should be replaced by “T/E”. The corrected figure 4 is presented below.

Fig. 4 Flow chart of the study (Corrected)


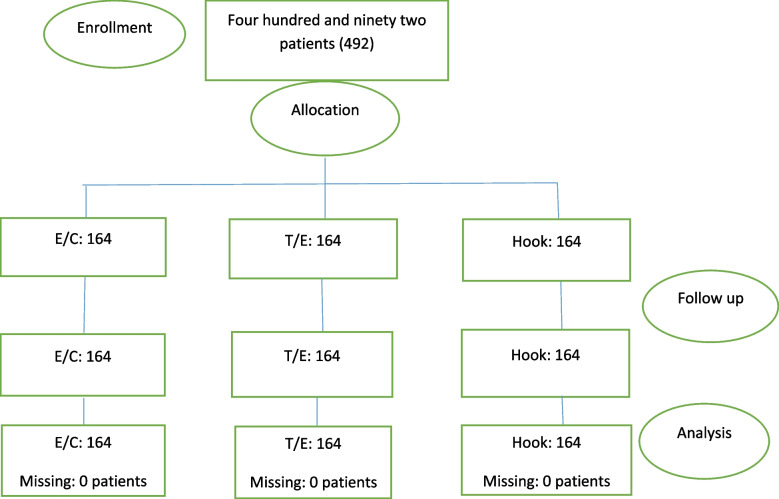


In the method section, line 17, paragraph 4: “Increase ETCO2 to more than 20 mmHg 2O and return to baseline [6].” The “2O” should be deleted.

The original article [1] has been updated.
